# A Novel Technique for Intraoral Ultrasound-Guided Aspiration of Peritonsillar Abscess

**DOI:** 10.3390/diagnostics8030050

**Published:** 2018-08-02

**Authors:** Tobias Todsen, Mads Georg Stage, Christoffer Holst Hahn

**Affiliations:** 1 Department of Otorhinolaryngology, Head and Neck Surgery & Audiology, Rigshospitalet University Hospital, 2100 Copenhagen, Denmark; Mads.Georg.Stage.01@regionh.dk (M.G.S.); Christoffer.Holst.Hahn.01@regionh.dk (C.H.H.); 2 Department of Otorhinolaryngology and Maxillofacial Surgery, Zealand University Hospital, 4600 Køge, Denmark

**Keywords:** point-of-care ultrasound, intraoral ultrasound, peritonsillar abscess, ultrasound-guided aspiration

## Abstract

Peritonsillar abscess (PTA) is a common complication to acute tonsillitis. The treatment is drainage of the abscess, but many needle aspirations are unsuccessful due to a low diagnostic accuracy based on oral examination only. In this article, we describe how intraoral ultrasound can be added to improve the diagnostic work-up of PTA and present a novel technique for ultrasound-guided aspiration of PTA, using a small pencil-shaped transducer. We present our first clinical experiences with this technique and describe how it could be integrated in a clinical setting to guide safe and successful needle aspirations of PTA.

## 1. Introduction

**Figure 1:**
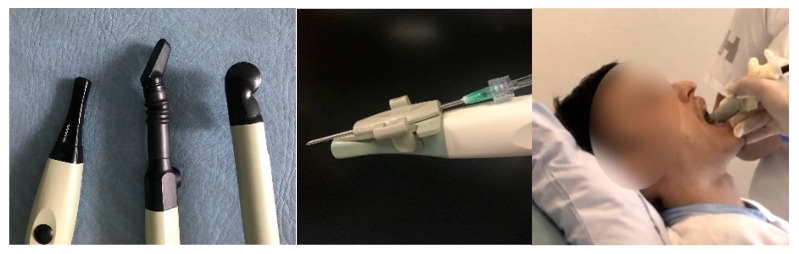
Different types of transducers that can be used for intraoral ultrasound (**Left**). A Burr-Hole, a Hockey Stick and a transvaginal/rectal transducers were from BK Ultrasound (Analogic, Peabody, MA, USA). A needle guide attached to the Burr-Hole transducer (**Middle**). An intraoral ultrasound examination conducted with a Burr-Hole transducer with a cover (**Right**).

Peritonsillar abscess (PTA) is a common deep infection in relation to the palatine tonsil with an incidence of 30 cases per 100,000 people per year in the United States [1]. Early treatment, in form of drainage of the pus, is important to avoid spreading into the surrounding tissue and fatal complications [2]. The formation of an abscess is often preceded by an acute tonsillitis that progresses to a peritonsillar cellulitis and further to a PTA. The patients typically complain about unilateral sore throat, fever, ipsilateral ear pain and decreased oral intake [3]. The oral examination may find muffled voice, trismus and unilateral erythematous and bulging palate with the corresponding tonsil displaced to the midline or beyond. Most patients with PTA can be treated in outpatient clinic by an otolaryngologist or an emergency physician with needle aspiration (or incision) using local anesthetic [1,4]. The landmark technique is traditional used to determine the point with the maximum bulging and fluctuance—usually in the superior pole of the tonsil—where the needle is inserted for “blind” aspiration [3,5]. If the aspiration is unsuccessful, further attempts will be conducted typical in the middle and lower poles of the tonsil [6]. However, the diagnostic accuracy of PTA, based on physical examination only, is low (sensitivity of 78% and specificity of 50%) [7], and may lead to many unnecessary attempts at drainage of peritonsillar cellulitis with no therapeutic effect [8]. Computed tomography (CT) with contrast has a high sensitivity for PTA, but is expensive and exposes young patients to ionizing radiation. Instead, intraoral ultrasound can provide ionized-free, low-cost and real-time imaging of PTA, though it may be difficult to use in patients with severe trismus and active oral tongue musculature [9,10]. Most studies only use intraoral ultrasound as a static diagnostic image modality, and afterwards, perform a “blind” needle aspiration of the PTA, as a two-step maneuver [7,9,11,12,13,14,15,16,17]. A few case reports describe the use of an endocavity transducer, designed for transvaginal examination for real-time image guidance of the PTA needle aspiration [18,19,20]. However, the size of the endocavity transducer makes it difficult to handle in the oral cavity without triggering the gag reflex of the patients. Instead, we have developed a novel technique using a smaller pencil-shaped transducer (originally developed for neurosurgical imaging through a burr hole in the skull) for intraoral ultrasound-guided aspiration of PTA (see Figure 1). We will in this article describe our intraoral ultrasound approach and present some illustrative images from clinical practice. Further, we will present a case report and discuss the potential impact of care for patients with PTA.

## 2. Materials and Methods

**Figure 2:**
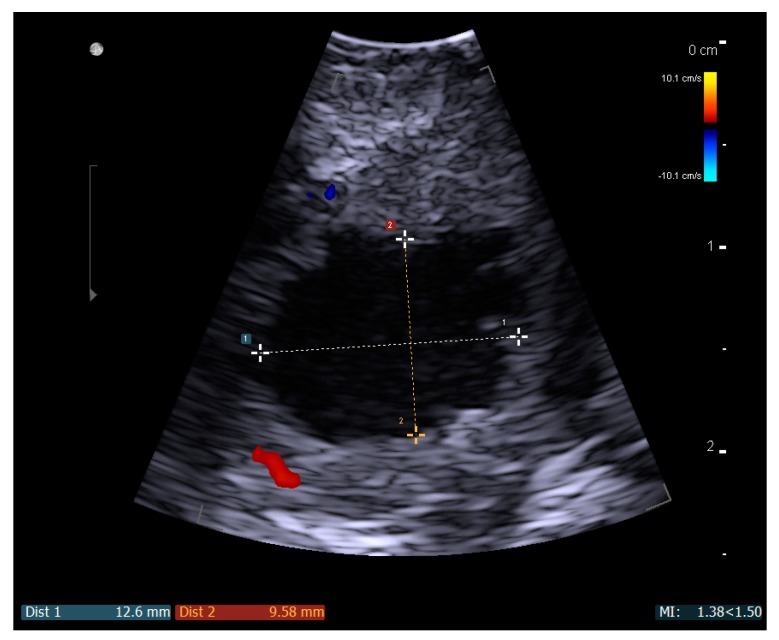
A static ultrasound image with a Burr-Hole N11C5s BK Ultrasound transducer of a left-side peritonsillar abscess seen as the measured hypoechoic area.

**Figure 3:**
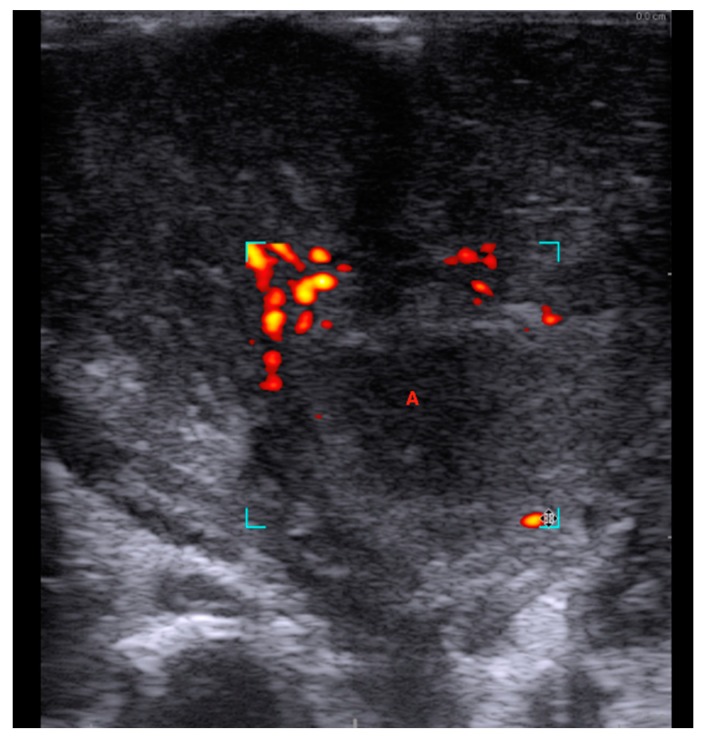
Power Doppler demonstrated no vascular activity in the ill-defined hypoechoic area (A), supporting the diagnose of a peritonsillar abscess. Ultrasound image with a Hockey Stick 8809 transducer and a Flex Focus 800 BK Ultrasound machine (Analogic, Peabody, MA, USA).

**Figure 4:**
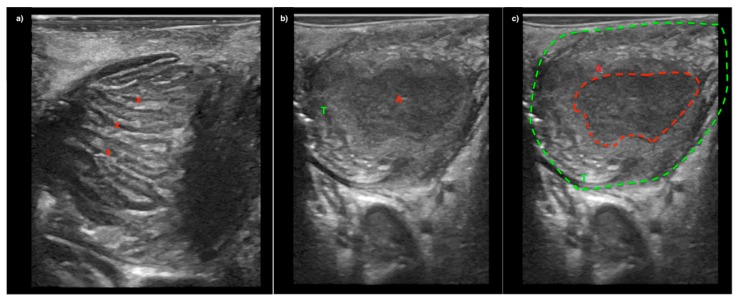
Image (**a**) A patient with severe tonsillitis presenting with peritonsillar swelling. Tonsillar hypertrophy is seen on ultrasound with inflamed tonsillar crypts (x) but without abscess. Image (**b**) and (**c**) Ultrasound image from another patient showing the palatine tonsil (T) with an abscess cavity (A). All imaged were captured with a Hockey Stick transducer and a GE Logiq S7 Ultrasound System (GE_Healthcare Chicago, Chicago, IL, USA).

**Figure 5:**
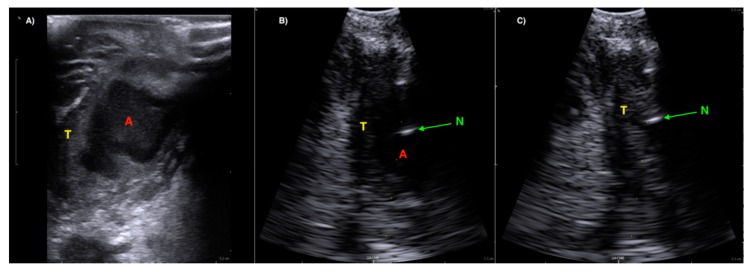
(Image (**A**)) A well-defined hypoechoic peritonsillar abscess (A) seen with a linear Hockey Stick transducer (BK Ultrasound) in relation to the Palatine Tonsil (T). (Image (**B**)) Ultrasound image from the same patient using a convex array Burr-Hole transducer with needle guide. The tip of the needle is seen as a hypoechoic reflection (N) in the abscess cavity (A). (Image (**C**)) Ultrasound image after successful aspiration from the abscess cavity emptied of pus.

Topical anesthetic (lidocaine, 10 mg/dose) should be sprayed to the posterior pharynx in order to decrease the gag reflex before intraoral ultrasound is conducted. A pencil-shaped Burr-Hole 8863 transducer (BK Ultrasound, Peabody, MA, USA) is suitable for intraoral ultrasound with the small curved head placed on the edematous palatoglossal arch and swiped from the cranial to caudal end of the tonsil. An abscess cavity can be seen as a hypoechoic area in relation to the tonsil (see Figure 2). If there are any doubts about the presence of an abscess, Power Doppler should be used (Figure 3) or ultrasound of the opposite tonsil should be performed as a reference. Further, a linear Hockey Stick transducer can also be used for improved intraoral imaging to help differentiate severe tonsillitis/cellulitis from a PTA (see Figure 4). Local anesthesia (e.g., 2% lidocaine with 5 microgram epinephrine) should be infiltrated in the mucous membrane if a PTA is confirmed. A needle guide is attached to the Burr-Hole transducer with an on-screen ultrasound needle guideline to ensure that small and deep PTAs are precise and safely drained. When the needle tip is visualized into the abscess cavity, the assisting nurse can aspirate the pus into a syringe until the hypoechoic area disappear on the ultrasound image (see Figure 5). A larger PTA cavity can also be aspirated with a free-hand ultrasound-guided technique with use of a syringe holder for aspirating without the help from an assistant. A pean or knife might be used to open the abscess cavity for further drainage after aspiration. The patient—who is not airway compromised—can now be discharged with oral antibiotics and painkillers for follow-up in the outpatient clinic.

## 3. Results

**Figure 6:**
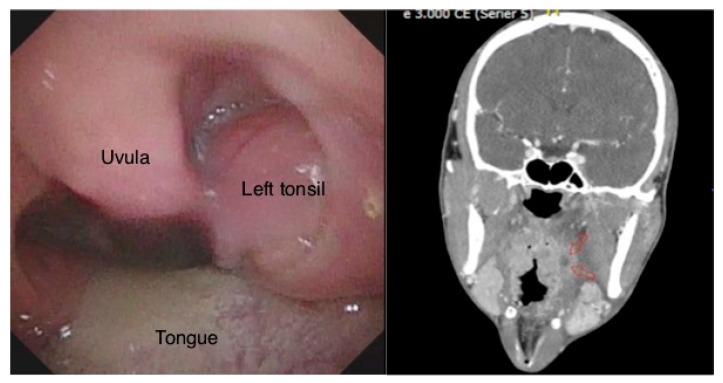
The oral examination with left-side peritonsillar swelling (**Left**). A deep left-side peritonsillar abscess, indicated by red arrows in the computed tomography (CT) image (**Right**).

A young man in his early 30s was referred to the Department of Otorhinolaryngology, Head and Neck Surgery & Audiology, Rigshospitalet with suspicion of a PTA by the emergency department. The initial clinical exam confirmed the suspicion of left-side PTA, and three blind needle aspiration attempts were performed using the traditional landmark technique without aspiration of pus. A CT examination with contrast was ordered and a deep abscess in relation to the left palatine tonsil was found (see Figure 6). The patient was therefore planned for an acute tonsillectomy in general anesthesia (Quincy tonsillectomy) to ensure drainage of the abscess cavity. However, due to other emergency surgical procedures, the operation was postponed. Instead, ultrasound-guided needle aspiration, as described in the method section, was successfully performed with local anesthetic (see Video S1, Supplementary). Afterwards, the patient was discharged with oral antibiotics and follow-up in the outpatient clinic.

## 4. Discussion

In this article, we described a new technique for point-of-care intraoral ultrasound of the palatine tonsils. We presented its use in a case with a small deep PTA, where aspiration with traditional landmark technique was unsuccessful, but ultrasound-guided needle aspiration succeeded instead. We used a new small pencil-shaped transducer that allowed for visualization of the tonsil, palatoglossal arch and decreased patient discomfort. Compared to other studies, our technique allows real-time needle guidance to ensure safe and complete drainage of the abscess cavity, which is also suitable for patients with trismus. We believe this technique can be used to decrease the number of unsuccessful needle aspirations compared to the landmark technique, while the real-time ultrasound guidance can ensure precise needle incision and avoid damage to vascular structures. We only recommend the intraoral ultrasound technique performed on adults [21], while transcutaneous cervical ultrasound is better tolerated for children and should be preferred for these cases instead [22]. Another limitation of our technique is the use of a special neurosurgical transducer, which is not traditionally used in Emergency Medicine or Otolaryngology. A linear Hockey Stick transducer is more commonly available and will actually provide better image resolution of the tonsils due to more transducer crystals and higher frequency, compared to the Burr-Hole transducer (see Figure 5) [23]. Most ultrasound manufacturers have Hockey Stick transducers available, and we have good experiences with equipment from both GE (GE_Healthcare Chicago, Chicago, IL, USA) and BK Ultrasound (Analogic, Peabody, MA, USA) in our departments. The GE transducer provides the best image quality of the tonsils (see Figure 3), while the Hockey Stick transducer from BK has a flexible tip, making it very suitable for intraoral use. However, due to the size of the Hockey Stick transducer, we could use it as a static imaging modality, while the needle incision afterwards is performed “blind” [17]. The intraoral ultrasound examinations illustrated in this article were all obtained by Tobias Todsen, who is an experienced ultrasound resident in Otolaryngology, and certified in head and neck ultrasound. However, point-of-care ultrasound is a very user-dependent image modality, requiring both technical and image interpretation skills by the physician [24,25]. It is therefore unknown if our initial results can be generalized to other settings with physicians’ without intraoral ultrasound experience, and further studies are needed to assess the learning curves [26,27].

This article describes a new method for ultrasound-guided aspiration of PTA, which may decrease patient discomfort and the number of unsuccessful needle aspiration attempts. However, we only described our initial clinical experiences with this technique and future randomized controlled trials are needed to explore the patient outcome and cost-effectiveness. 

## Supplementary Materials

The following are available online at http://www.mdpi.com/2075-4418/8/3/50/s1.
